# Pericardial Conundrum: Unmasking Tuberculosis as the Culprit

**DOI:** 10.1016/j.jaccas.2025.104428

**Published:** 2025-07-30

**Authors:** Brittni McClellan, Brandon A. Grodman, Jessica A. LaVoie, Nathan J. Foster, Souheil E. Saba, Michael W. Lee

**Affiliations:** a Cardiology Department, Henry Ford Providence Hospital, Southfield, Michigan, USA; b Internal Medicine Department, Henry Ford Providence Hospital, Southfield, Michigan, USA; c Cardiothoracic Surgery Department, Henry Ford Providence Hospital, Southfield, Michigan, USA

**Keywords:** constrictive, echocardiography, hemodynamics, imaging, pericardial effusion

## Abstract

**Background:**

Tuberculosis, caused by *Mycobacterium tuberculosis*, primarily affects the lungs but can involve other organs, termed extrapulmonary tuberculosis. Tuberculous pericarditis (TBP) is a rare form, representing approximately 1% of tuberculosis-related autopsies and 4% of acute pericarditis cases in developed countries.

**Case Summary:**

A 29-year-old healthy Indian man presented with fever, night sweats, and weight loss. Imaging revealed a large pericardial effusion with tamponade physiology. He underwent pericardiocentesis and a surgical pericardial window, with biopsy confirming *M. tuberculosis*. He was treated with rifampin, isoniazid, pyrazinamide, and ethambutol therapy, colchicine, and a steroid taper, resulting in clinical improvement.

**Discussion:**

TBP is rare in developed regions and presents diagnostic challenges because of nonspecific symptoms and delayed culture results. Early recognition and intervention are critical to prevent progression to constrictive pericarditis and improve outcomes.

**Take-Home Message:**

A high index of suspicion for TBP is essential in patients with pericardial effusion to enable timely diagnosis and intervention, optimizing clinical outcomes.

## Graphical abstract

**Figure:**
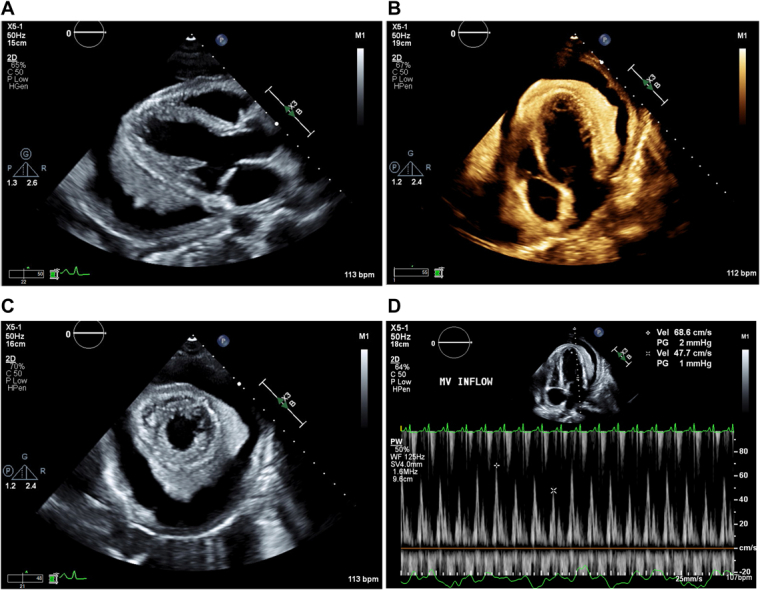

## History of Presentation

A 29-year-old previously healthy man of Indian descent, who migrated to the United States 3 years ago, presented to the emergency department with a 2-week history of fever, peaking at 102 °F, productive cough, night sweats, unintentional weight loss, and a single episode of loose stools the night before admission. The patient denied recent international travel. On arrival, he was tachycardic with heart rate sustaining 120 beats/min and tachypneic with respiratory rate 25 breaths/min. Physical examination was otherwise unremarkable.

## Past Medical History

The patient was an Indian migrant with no significant past medical history.

## Differential Diagnosis

The initial differential diagnosis was broad due to the patient's systemic symptoms, with considerations including viral, bacterial, or mycobacterial infections, as well as a potential neoplastic process such as lymphoma. Additional laboratory testing, including pericardiocentesis, revealed an exudative pericardial effusion, which narrowed the differential diagnosis to tuberculous pericarditis (TBP), purulent pericardial effusion due to bacterial infection, malignancy, and hemopericardium, with the leading diagnosis favoring TBP.

## Investigations

Initial laboratory test results showed a white blood cell count of 5,000/μL, a hemoglobin level of 11.8 g/dL, a mean corpuscular volume of 76.1 fL, a platelet count of 342,000/μL, and an alkaline phosphatase level of 168 U/L. The high-sensitivity troponin level was <6 ng/L, the C-reactive protein level was 57.9 mg/L, and the erythrocyte sedimentation rate was 31 mm/h. The glucose level was 78 mg/dL and the total protein level was 7.8 g/dL. A chest radiograph revealed cardiomegaly, raising concern for pericardial effusion, confirmed by point-of-care ultrasound. The patient was admitted to the intensive care unit for monitoring and a formal echocardiogram.

**Figure 1:**
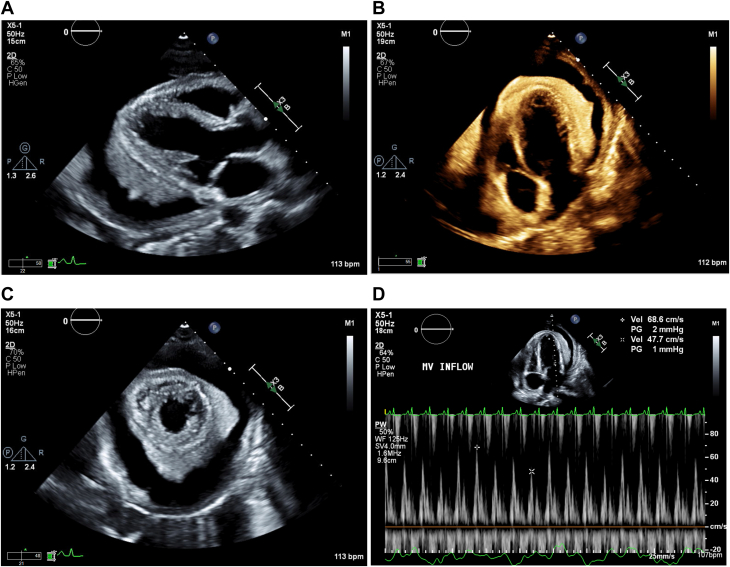
Initial Transthoracic Echocardiogram on the Patient's Presentation to the Hospital Multiple windows demonstrate the large pericardial effusion with fibrinous material collection adherent to the cardiac surface. (A) Parasternal long axis. (B) Apical 4-chamber view. (C) Parasternal short axis. (D) Mitral inflow demonstrating an abnormal respiratory variation of 30.47%, consistent with hemodynamic compromise.

**Figure 2:**
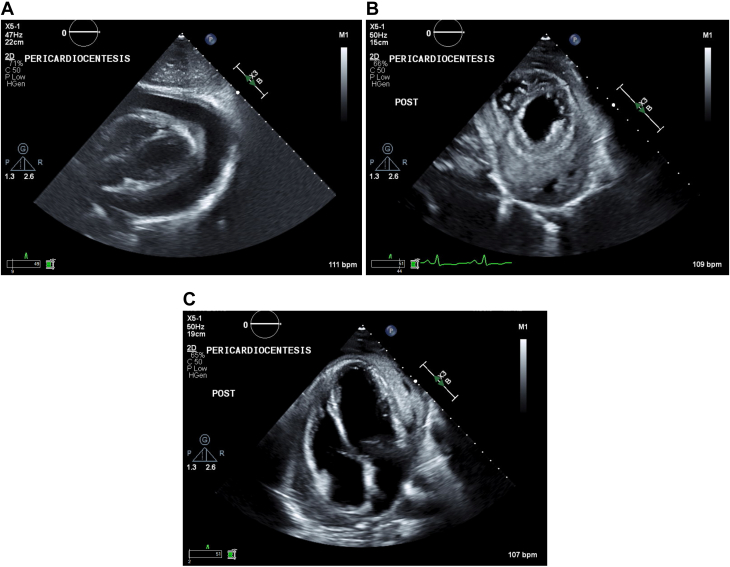
Periprocedural Echocardiography Transthoracic echocardiogram imaging obtained (A) immediately before pericardiocentesis and (B, C) immediately after pericardiocentesis and drain insertion, with visualization of remaining complex fluid collection.

The echocardiogram showed a large circumferential pericardial effusion with extensive fibrinous material adherent to the cardiac surface and evidence of hemodynamic compromise with an abnormal variation of mitral inflow of 30.47% (Figure 1). The patient underwent urgent pericardiocentesis and drain placement, removing 550 mL of tea-colored fluid without complications. A follow-up echocardiogram (Figure 2) showed only a trivial effusion. Pericardial fluid analysis revealed <2,000 RBCs, 1,549 WBCs (64% polymorphonuclear neutrophils, 32% lymphocytes), a glucose level of <2 mg/dL, a lactate dehydrogenase level of 1,409 U/L, and a protein level of 5.6 g/dL. The adenosine deaminase level was elevated at 40 U/L (normal range: 0-40 U/L).

Antibody titers for all Coxsackie A and B serotypes were positive, but interpreted as an anamnestic response; viral myocarditis was considered unlikely because of the low pericardial glucose. Given the fluid analysis, demographics, and organized effusion, clinical suspicion for TBP remained high, and additional cultures and studies were sent for analysis.

**Figure 3:**
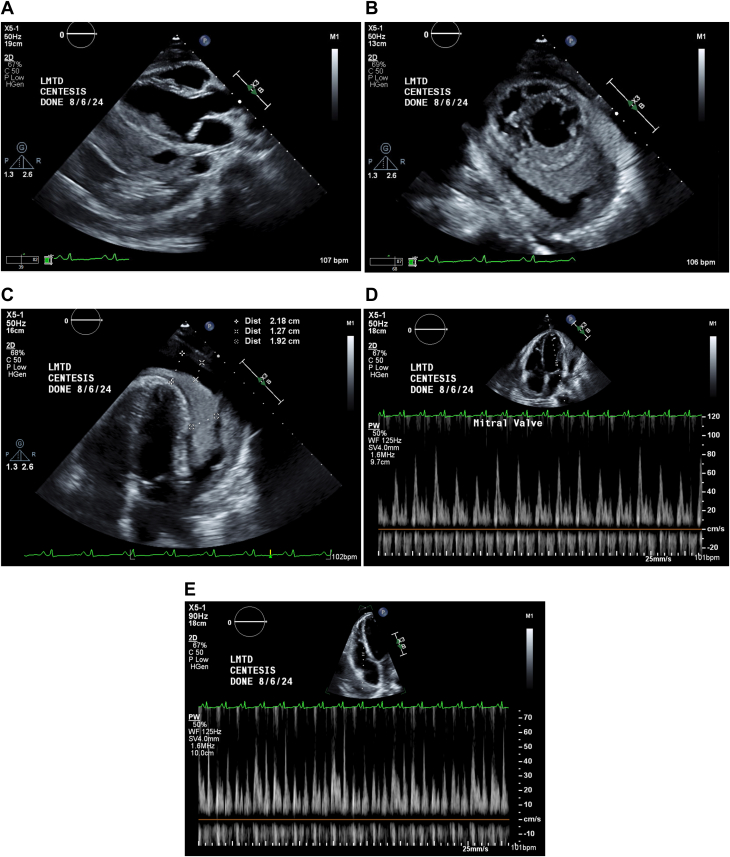
Postprocedural Echocardiography Repeat transthoracic echocardiogram imaging day 1 after pericardiocentesis and drain placement (A-C) with multiple windows displaying persistent complex fluid collection concerning for hemodynamic comprise (D, E) despite adequate placement and function.

On postprocedural day 1, the echocardiogram showed ventricular septal bounce (Video 1), persistent large organized pericardial effusion, and complex left pleural effusion (Figure 3). Findings suggested ineffective drain function. Given ongoing clinical decline, a surgical pericardial window was pursued for drainage and a biopsy for histopathology, DNA amplification, and cultures.

## Management

**Figure 4:**
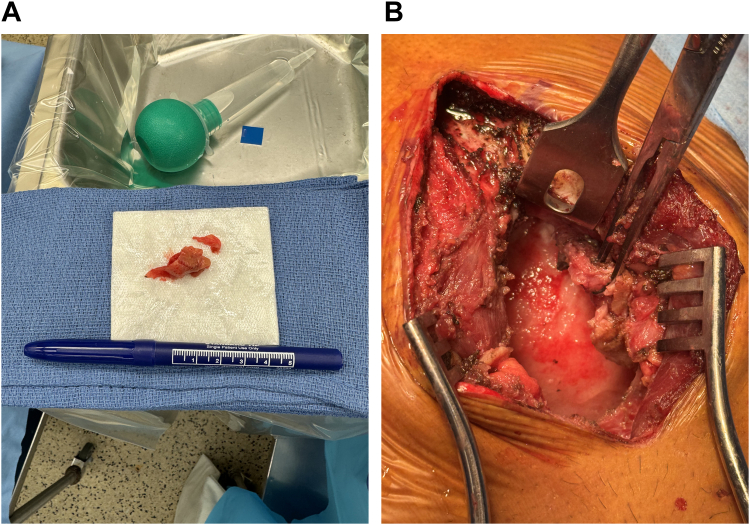
Operative Findings Gross visualization of intraoperative extended pericardial window including (A) the fibrinous material adherent to and (B) excised from the cardiac surface.

**Figure 5:**
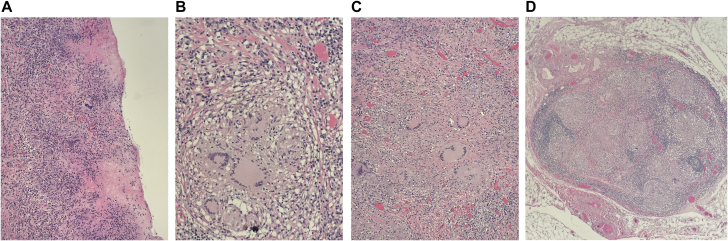
Histopathology of Pericardial Biopsy and Associated Lymph Node Biopsies (A) Necrotizing granulomatous inflammation of the pericardium. (B) Giant cell located within the pericardium. (C) Granulomatous lymphadenitis with visualized giant cells. (D) Lymph node and its capsule in its entirety, redemonstrating granulomatous lymphadenitis with loss of normal lymph node architecture.

The patient underwent an extended pericardial window with a biopsy of preperitoneal fat and pericardial tissue. One hundred milliliters of free-flowing fluid was drained, and well-organized pericardial tissue was visualized. Blunt dissection with ring forceps revealed a fibrous rind on the heart, adherent and difficult to strip off the cardiac surface (Figure 4). Enlarged lymph nodes in the preperitoneal and anterior mediastinal/pericardial fat were biopsied. Fluid samples collected during this procedure were positive for acid fast bacilli, confirmed as *Mycobacterium tuberculosis* with polymerase chain reaction. Surgical pathology of the biopsied pericardium revealed necrotizing granulomatous inflammation and associated giant cells (Figures 5A and 5B), and a biopsy of the associated lymph nodes revealed granulomatous lymphadenitis (Figures 5C and 5D). Of interest, stains for acid fast bacilli and fungal microorganisms were negative in both samples.

• Pericardiocentesis—Fluid analysis and cultures
• Extended pericardial window with pericardial biopsy

## Outcome and Follow-Up

The patient made a favorable recovery after the pericardial window. He was treated with a regimen of rifampin, isoniazid, pyrazinamide, and ethambutol (RIPE therapy) for 2 months, followed by 4 months with rifampin and isoniazid alone, along with continued vitamin B6. In addition, the patient was started on colchicine and a steroid taper for the management of constrictive pericarditis, both of which were discontinued at the patient's 1-month cardiology follow-up appointment. His treatment was also monitored by the County Health Department.

## Discussion

This case discusses the rare but potentially serious complication of TBP in an immunocompetent patient in a developed country. As previously noted, TBP has been found in approximately 1% of all autopsied cases of tuberculosis (TB) and in 1% to 2% of instances of pulmonary TB. It accounts for around 4% of pericarditis cases in developed countries.^1^^,^^2^ One of the more challenging aspects of extrapulmonary TB, and specifically TBP, is establishing the route of invasion. As such, understanding the pathogenesis of TBP is crucial. Most documented cases of TBP are due to retrograde lymphatic spread of *M. tuberculosis* from peritracheal, peribronchial, or mediastinal lymph nodes, or by hematogenous spread after primary TB infection.^1^ This pathogenesis is important when evaluating clinical presentation, which can vary depending on the stage of the disease. Early presentations often manifest with insidious onset with fever of unknown origin, malaise, and weakness. As the disease progresses, further symptoms can include dyspnea, cough, weight loss, and heart failure symptoms, which are described in approximately 40% to 70% of patients.^2^

TBP is recognized to have 4 pathologic stages, which are important for both diagnosis and management. The first stage, known as the dry stage, is characterized by fibrinous exudation, initial polymorphonuclear leukocytosis, relatively abundant mycobacteria, and early granuloma formation with loose organization of macrophages and T cells. The second stage, called the effusive stage, involves a serosanguineous effusion with a predominantly lymphocytic exudate, along with monocytes and foam cells. In the third stage, or absorptive stage, the effusion is absorbed, and granulomatous caseation and pericardial thickening occur due to fibrin, collagen deposition, and ultimately fibrosis. The final stage, the constrictive stage, is marked by fibrosing of the visceral and parietal pericardium, which contracts on the cardiac chambers, potentially becoming calcified and encasing the heart in a fibrocalcific rind. This constriction impedes diastolic filling, causing the classic symptoms and signs associated with constrictive pericarditis.^1^^,^^2^

Clinically, TBP can manifest in 1 of 3 forms: pericardial effusion, constrictive pericarditis, or a combination of both. If the disease presents acutely, as it does in approximately 20% of patients, individuals may exhibit tachycardia, raised jugular venous pressure with a blunt Y descent, pulsus paradoxus, distant heart sounds, pericardial rub, and hemodynamic instability, which are concerning for cardiac tamponade. Many of these findings were evident in our patient.

Despite advances in diagnostic techniques, up to 15% to 20% of pericardial diseases remain undiagnosed.^3^ In TB pericardial disease, one of the most widely used biochemical tests is adenosine deaminase. Of note, its sensitivity as a diagnostic rule-out test is limited. For this reason, pericardiocentesis remains essential, providing both therapeutic and diagnostic benefits. Pericardial fluid obtained through this procedure is typically exudative (based on Light's criteria), protein-rich, lymphocytic, and often macroscopically bloodstained.^3^

Pericardial fluid culture is still considered the most reliable diagnostic test for TBP, with sensitivity ranging between 53% and 75%. However, cultures can take up to 3 weeks to yield results. Unfortunately, there have been limited advancements in the technology used to enhance the diagnostic yield of pericardial fluid smear for acid-fast bacilli, and microscopy remains the standard method. This has led to continued reliance on pericardial fluid culture despite its prolonged turnaround time and reduced yield.

Given the time required to achieve definitive diagnostic results and the inability to provide rapid confirmation, many practitioners in high TB-burden regions often resort to empiric treatment for suspected TBP. Of concern, observational evidence suggests that empiric therapy, while often necessary in resource-limited settings, is associated with increased morbidity and mortality.^3^ For this reason, several authors have proposed diagnostic criteria for “definitive” and “probable” TBP, based on a combination of clinical and laboratory findings to help guide management decisions in these challenging cases.

The case we present exemplifies the diagnostic complexity and clinical challenges in managing TBP, especially when it occurs in immunocompetent individuals. Early suspicion, appropriate diagnostic testing, and timely therapeutic intervention remain crucial to reducing the potential for severe complications such as constrictive pericarditis and cardiac tamponade.

## Conclusions

TBP is a rare but serious complication of TB that can occur even in immunocompetent individuals, especially in regions of endemic TB. Early diagnosis and intervention are essential to prevent life-threatening complications. This case highlights the need for increased awareness of TBP, prompt evaluation, and early therapeutic intervention to ensure favorable outcomes.

**Visual Summary:** Tuberculous Pericarditis: Key Clinical Data

Day/Event	Clinical Findings/Action	Key Results/Interpretation
Presentation	29-year-old Indian man with 2-week fever, cough, night sweats, weight loss, and 1 episode of diarrhea	Vitals: HR 120 beats/min, RR 25 breaths/min. No other significant examination findings
Initial labs and imaging	CRP 57.9 mg/L, ESR 31 mm/h, WBC 5,000/μL, Hb 11.8 g/dL, CXR → cardiomegaly	Suspected pericardial effusion; admitted to ICU
Echocardiogram	Large pericardial effusion, fibrinous organization, and signs of tamponade	Urgent pericardiocentesis performed
Pericardiocentesis	550 mL tea-colored fluid drained; WBC 1,549 (64% PMNs), glucose <2 mg/dL, and protein 5.6 g/dL	Tuberculous pericarditis suspected based on profile + patient demographic
Postprocedure echo	Reaccumulation of effusion, constrictive signs, and complex pleural effusion	Drain deemed inadequate—worsening clinical status
Surgical intervention	Extended pericardial window + biopsies (pericardium, preperitoneal, and mediastinal fat/lymph nodes)	Gross fibrous rind, necrotizing granulomatous inflammation seen
Fluid/tissue results	Pericardial fluid: AFB positive, *Mycobacterium tuberculosis* confirmed Biopsies: granulomatous inflammation	Histopathology consistent with tuberculous pericarditis
Treatment initiated	RIPE therapy + vitamin B6, colchicine, and a steroid taper for constrictive physiology	Favorable recovery

AFB = acid-fast bacilli; CRP = C-reactive protein; CXR = chest x-ray; ESR = erythrocyte sedimentation rate; Hb = hemoglobin; HR = heart rate; ICU = intensive care unit; PMN = polymorphonuclear neutrophil; RIPE = rifampin, isoniazid, pyrazinamide, and ethambutol; RR = respiratory rate; WBC = white blood cells.

## Ethics Approval

This submission is a retrospective case report and, as such, did not require formal ethical/IRB approval according to our institution's guidelines.

**Take-Home Messages**

• This case highlights the pathogenesis, clinical course, and therapeutic strategies required for the management of tuberculous pericarditis.
• It emphasizes the criticalness of early recognition and diagnosis in order to avoid dreaded complications of pathophysiology.

## Funding Support and Author Disclosures

The authors have reported that they have no relationships relevant to the contents of this paper to disclose.

## Appendix

Video 1 Repeat Transthoracic Echocardiogram Imaging Day 1 After Pericardiocentesis and Drain Placement Showing Ventricular Septal Bounce
